# Prognosis stratification and response to treatment in breast cancer based on one-carbon metabolism-related signature

**DOI:** 10.3389/fonc.2023.1288909

**Published:** 2024-01-04

**Authors:** Tongxin Zhang, Jingyu Liu, Meihuan Wang, Xiao Liu, Jia Qu, Huawei Zhang

**Affiliations:** Department of Ultrasound, Shandong Provincial Hospital Affiliated to Shandong First Medical University, Jinan, Shandong, China

**Keywords:** one-carbon metabolism, breast cancer, prognosis, immune cell infiltration, immunotherapy, drug sensitivity

## Abstract

**Introduction:**

Breast cancer (BC) is the most common malignant tumor in the female population. Despite staging and treatment consensus guidelines, significant heterogeneity exists in BC patients' prognosis and treatment efficacy. Alterations in one-carbon (1C) metabolism are critical for tumor growth, but the value of the role of 1C metabolism in BC has not been fully investigated.

**Methods:**

To investigate the prognostic value of 1C metabolism-related genes in BC, 72 1C metabolism-related genes from GSE20685 dataset were used to construct a risk-score model via univariate Cox regression analysis and the least absolute shrinkage and selection operator (LASSO) regression algorithm, which was validated on three external datasets. Based on the risk score, all BC patients were categorized into high-risk and low-risk groups. The predictive ability of the model in the four datasets was verified by plotting Kaplan-Meier curve and receiver operating characteristic (ROC) curve. The candidate genes were then analyzed in relation to gene mutations, gene enrichment pathways, immune infiltration, immunotherapy, and drug sensitivity.

**Results:**

We identified a 7-gene 1C metabolism-related signature for prognosis and structured a prognostic model. ROC analysis demonstrated that the model accurately predicted the 2-, 3-, and 5-year overall survival rate of BC patients in the four cohorts. Kaplan-Meier analysis revealed that survival time of high-risk patients was markedly shorter than that of low-risk patients (p < 0.05). Meanwhile, high-risk patients had a higher tumor mutational burden (TMB), enrichment of tumor-associated pathways such as the IL-17 signaling pathway, lower levels of T follicular helper (Tfh) and B cells naive infiltration, and poorer response to immunotherapy. Furthermore, a strong correlation was found between MAT2B and CHKB and immune checkpoints.

**Discussion:**

These findings offer new insights into the effect of 1C metabolism in the onset, progression, and therapy of BC and can be used to assess BC patients' prognosis, study immune infiltration, and develop potentially more effective clinical treatment options.

## 1 Introduction

As recently reported, the incidence of breast cancer (BC) continues to rise, accounting for 31% of all new cancer cases among the female population in the U.S. in 2023 (1). Currently, BC treatment mainly includes chemotherapy, radiotherapy, targeted therapy, immunotherapy, and preoperative and postoperative endocrine therapy, according to international consensus guidelines (2). However, due to tumor heterogeneity, metastatic heterogeneity, and drug resistance, many BC patients still do not benefit from chemotherapy, endocrine therapy, and other routine treatments and have poor prognoses (3, 4). After diagnosis and routine treatment of the primary tumor, 20%-30% of BC patients may develop metastases (4), and metastatic BC has been reported to have a 5-year survival rate of only 28% (5). Thus, the search for new tumor biomarkers and therapeutic targets is crucial for identifying BC patients with poor prognoses and guiding the treatment of BC.

One-carbon (1C) metabolism involves a range of interrelated metabolic pathways such as the methionine cycle, the folate cycle, and the transsulfuration pathway, which are essential for cellular function and facilitate the distribution of 1C units to different cellular processes through a range of chemical reactions (6). These processes include cellular biosynthesis (DNA, amino acids, polyamines, phospholipids, and creatine, etc.), amino acid homeostasis (serine, glycine, and methionine), redox state maintenance, epigenetics regulation, and genome maintenance via regulation of nucleotide pools (7, 8). Importantly, in addition to the synthesis of nucleotides and certain amino acids, folate-mediated 1C metabolism controls the production of glutathione and S-adenosylmethionine, as well as other critical cellular processes associated with the rapid progression of malignancies (9). In addition, 1C metabolizing enzymes have been demonstrated to be up-regulated in expression in a variety of cancers (10). For example, SHMT2 has been determined to be overexpressed in BC, glioblastoma, and colorectal cancer (11–13). Elevated expression levels of SHMT2 in triple-negative breast cancer (TNBC) patients correlate with their poorer prognosis (14). Expression of DNMT3B in thyroid and hepatocellular carcinoma is closely related to their poor prognosis (15, 16). Today, certain drugs that target 1C metabolizing enzymes have been developed and applied in the clinic, including methotrexate and pemetrexed (8). These drugs have far-reaching implications in the treatment of many cancers, including BC (17–19).

Immune cells include cancer cells, non-tumor host cells (innate and adaptive immune cells, etc.), and their noncellular components, which are crucial players in the tumor microenvironment (TME) (20). Studies have shown that 1C metabolism affects immune cell function, especially T-cell activation (8). Tumor progression, invasion, metastasis, and drug resistance are emergent characteristics of tumor cell-TME interactions (21). Targeting the TME in combination with multiple therapeutic modalities, such as chemotherapy, radiation, immunotherapy, surgery, and nanotherapy, can synergistically and effectively target key pathways associated with disease pathogenesis (22). However, the specific effect of 1C metabolism on TME needs further study.

In this study, a 1C metabolism-related genes risk score model was constructed. Then the prognostic value of the seven candidate genes was confirmed by extensive analysis. Finally, the correlation of candidate genes with immune checkpoints, related immunotherapy, and sensitivity to common chemotherapeutic drugs was investigated to contribute to guiding the treatment of BC patients.

## 2 Materials and methods

### 2.1 Data source and processing

Gene expression profiles of 327 BC samples in the GSE20685 were obtained from Gene Expression Omnibus (GEO) database (https://www.ncbi.nlm.nih.gov/geo/) as a training dataset, together with their clinical data such as age, TNM stage, and survival status. Three additional GEO datasets were also obtained from GEO database as test datasets, including GSE88770, GSE58812, and GSE61304. The samples in dataset GSE58812 were all TNBC. After processing the datasets according to the same filtering criteria such as removing samples with incomplete data, expression profiles and clinical data were used to conduct subsequent analysis. Gene mutation data were acquired from The Cancer Genome Atlas (TCGA) database (https://portal.gdc.cancer.gov/). In addition, 72 1C metabolism-related genes were retrieved from the Molecular Signature Database (MSigDB, https://www.gsea-msigdb.org/gsea/msigdb) with the keyword “one-carbon metabolism” (**Supplementary Table 1**). These genes were used as the basis for our further studies.

### 2.2 Construction of the risk score model

We structured a risk signature assessment system based on the expression of 1C metabolism-related genes to analyze the correlation between these genes’ expression and BC prognosis. For this purpose, genes were binarized into high or low according to expression, and then raw expression was used for signature generation. Firstly, 1C metabolism-related genes with prognostic value in BC were extracted by univariate Cox regression analysis, and the genes with p < 0.05 were identified to be overall survival (OS)-related genes. Genes with non-significant differences in survival between high- and low-expression groups were removed by log-rank test. The least absolute shrinkage and selection operator (LASSO) regression (with R packages “glmnet”) was then used to determine non-zero coefficients, to achieve the purpose of eliminating potential predictors and selecting the optimal OS-related genes while preventing model overfitting. The LASSO regression tuned the model with 10-fold cross-validation. Additionally, we conducted multivariate Cox regression analysis to further determine model genes and risk coefficients. Finally, seven genes affecting prognosis were screened, including MAT2B, DNMT3B, AHCYL1, CHDH, SHMT2, CHKB, and CHPT1. For every patient, the product of coefficients and prognostic gene expression level was risk score. Furthermore, BC samples were categorized into two subgroups based on median risk scores, including high- and low-risk groups.

### 2.3 Prognostic model validation

For assessing the feasibility of the 1C metabolism-related genes risk score model, Kaplan-Meier survival analyses of OS were implemented between the high- and low-risk groups in the training set GSE20685, as well as the validation sets GSE88770 and GSE58812, respectively. Meanwhile, we used the outcome events and time in GSE61304 to validate the disease-free survival (DFS) of BC. In addition, the R package “timeROC” was used to plot receiver operating characteristic (ROC) curves of 2-, 3-, and 5-year survival. The area under the ROC curve (AUC) was also calculated to further analyze and validate the accuracy of the model.

### 2.4 Independent prognostic analysis and nomogram construction

Univariate and multivariate Cox regression analyses for age, TNM stage, and risk score were performed from the training dataset GSE20685 clinical information to assess independent prognostic factors for BC. Then, according to independent prognostic analyses, a nomogram combining the 1C metabolism-related risk score with other clinical characteristics in the training dataset was developed with the “rms” R package. In addition, we plotted calibration curves to visualize the consistency of the nomogram at 2, 3, and 5 years as evidence of its clinical predictive value. Additionally, we constructed a multivariate Cox model containing clinical characteristic information and risk scores on the training dataset, and then performed validation evaluations on the test datasets.

### 2.5 Functional enrichment analysis

To investigate potential biological functions and signaling pathways of the two groups, we employed the R package “Limma” for screening differentially expressed genes (DEGs) among both groups with the criteria of |log FC| > 1 and p-value_ t < 0.05, and then analyzed them with Gene Ontology (GO) and Kyoto Encyclopedia of Genes and Genomes (KEGG) analyses. Gene set enrichment analysis (GSEA) was performed on the training set using the R package “ClusterProfiler” to further identify the different biological processes involved in the two risk groups. The annotated gene set was extracted from the R package “org.Hs.eg.db” and used in our analysis.

### 2.6 Somatic mutations and immune infiltration analysis

We retrieved somatic mutation profiles of BC patients from TCGA database to analyze somatic mutations among both groups. The somatic mutation data were further analyzed with the R package “maftools”. Correlations between the expression of seven candidate genes and the tumor mutational burden (TMB) were analyzed, and the results were plotted by selecting those with significant correlations. Moreover, cell type identification by estimating relative subsets of RNA transcripts (CIBERSORT) was utilized to calculate the abundance of 22 tumor immuno-infiltrative cells within TME of BC samples from the GSE20685 cohort. Subsequently, differences in immune cell infiltration were analyzed for the two risk groups, with p-value < 0.05 considered statistically significant. The results were revealed by box plots.

### 2.7 Immune checkpoints analysis and immunotherapy response assessment

We analyzed the correlation of seven candidate genes with immunological checkpoint genes (ICGs). A list of 79 common ICGs can be obtained from a previous article (**Supplementary Table 2**) (23), and 71 of them were included in our expression matrix. Five ICGs significantly related to 1C metabolism-related genes (CD27, HLA-DPB1, HLA-E, CD40, and HLA-DMB) were screened to plot the correlation heat map. Furthermore, immunophenotype score (IPS) were obtained from The Cancer Immunome Atlas (TCIA, https://tcia.at/) database, which is helpful in screening patients who are sensitive to immune checkpoint inhibitors (ICIs). To match the sample in the IPS data, we used the TCGA-BRCA dataset for analysis. Hence, IPS differences between high and low expression of seven candidate genes were analyzed, and the results with statistically significant differences were selected to draw violin plots.

### 2.8 Drug sensitivity analysis

Response to chemotherapy in BC patients was assessed with the Genomics of Drug Sensitivity in Cancer database (GDSC, https://www.cancerrxgene.org) via the R package “oncoPredict”. Correlations between the two risk groups and the sensitivity to common chemotherapeutic drugs were calculated separately.

### 2.9 Statistical analysis

Statistical analyses were accomplished via R (version 4.2.2). Survival analyses were presented by Kaplan-Meier approach, and differences between groups were assessed with log-rank test. Pearson’s correlation test was used for correlation analysis. Univariate and multivariate analyses were performed with Cox regression models to determine independent risk factors. The p-value < 0.05 was regarded as statistically significant.

## 3 Results

### 3.1 Prognostic characteristics and value of 1C metabolism-related genes

**Figure 1:**
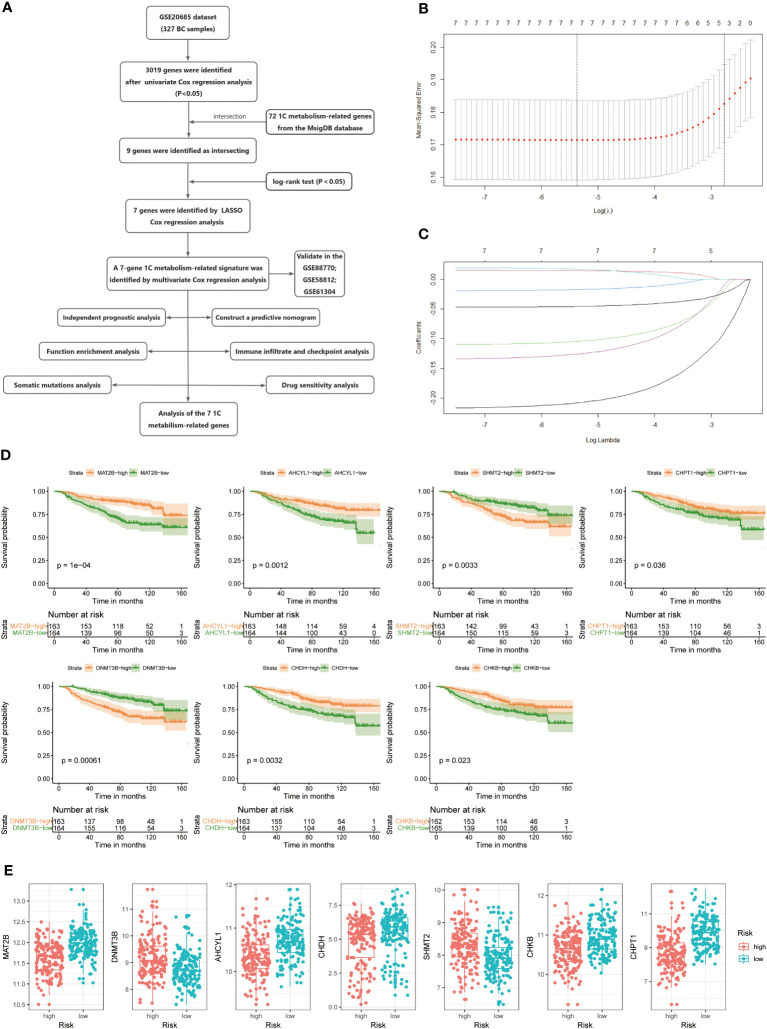
**(A)** Flow chart of the study. **(B, C)** LASSO Cox regression analysis to develop the prognostic model. **(D)** Kaplan-Meier survival curves of seven genes associated with OS. **(E)** Expression of seven genes in the high- and low-risk groups.

The study’s flow is illustrated in **Figure 1A**. We ultimately constructed a 7-gene 1C metabolism-related prognostic model, including MAT2B, DNMT3B, AHCYL1, CHDH, SHMT2, CHKB, and CHPT1. The process of LASSO regression was shown in **Figures 1B, C**. Kaplan-Meier analysis revealed seven genes correlated with OS. Among them, five genes, MAT2B, AHCY1, CHDH, CHKB, and CHPT1, were regarded as protective factors, whereas two other genes, DNMT3B and SHMT2, were considered as risk factors (p < 0.05) (**Figure 1D**). Meanwhile, the following risk score formula was obtained after multivariate Cox regression: risk score = (-1.166*MAT2B) + (-0.040*DNMT3B) + (-0.581*AHCYL1) + (-0.067CHDH) + (0.168*SHMT2) + (-0.609*CHKB) + (-0.346*CHPT1). Grouped according to the median risk score, the expression differences of the seven candidate genes in the two risk groups were presented in **Figure 1E**. The forest plot showed the results of stepwise multivariate Cox proportional hazards regression analysis (**Supplementary Figure 1**).

### 3.2 Validation of the 1C metabolism-related genes model prediction effect

**Figure 2:**
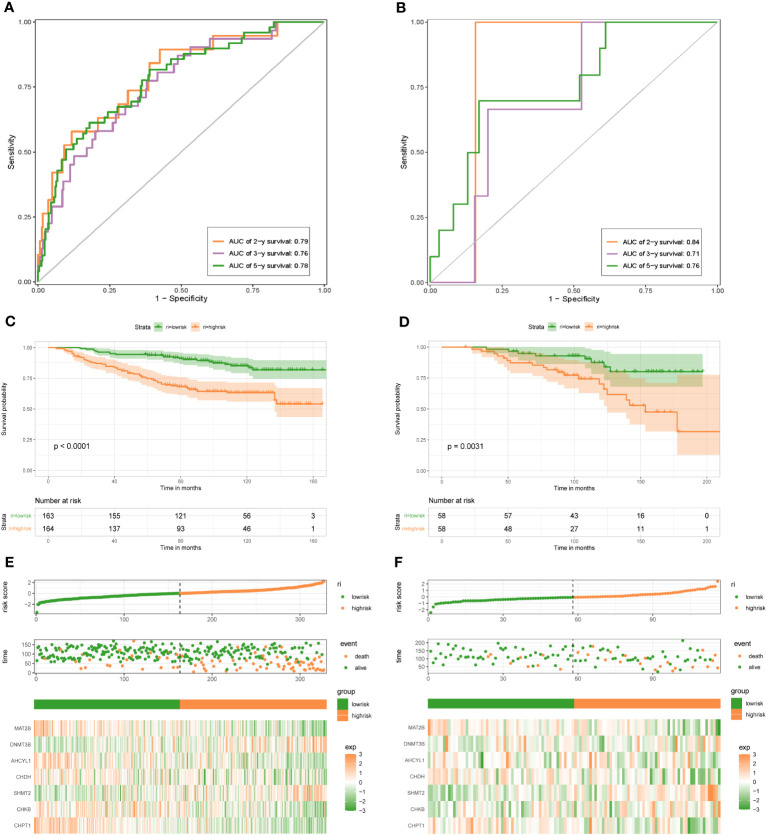
1C metabolism-related genes signature associated with the OS of BC patients. **(A)** The predictive value for the 2-y, 3-y, and 5-y OS in GSE20685 dataset. **(B)** The predictive value for the 2-y, 3-y, and 5-y OS in GSE88770 dataset. **(C)** The OS between the high- and low-risk groups in GSE20685 dataset. **(D)** The OS between the high- and low-risk groups in GSE88770 dataset. **(E)** The risk plot of the 1C metabolism-related genes signature in GSE20685 dataset. **(F)** The risk plot of the 1C metabolism-related genes signature in GSE88770 dataset.

**Figure 3:**
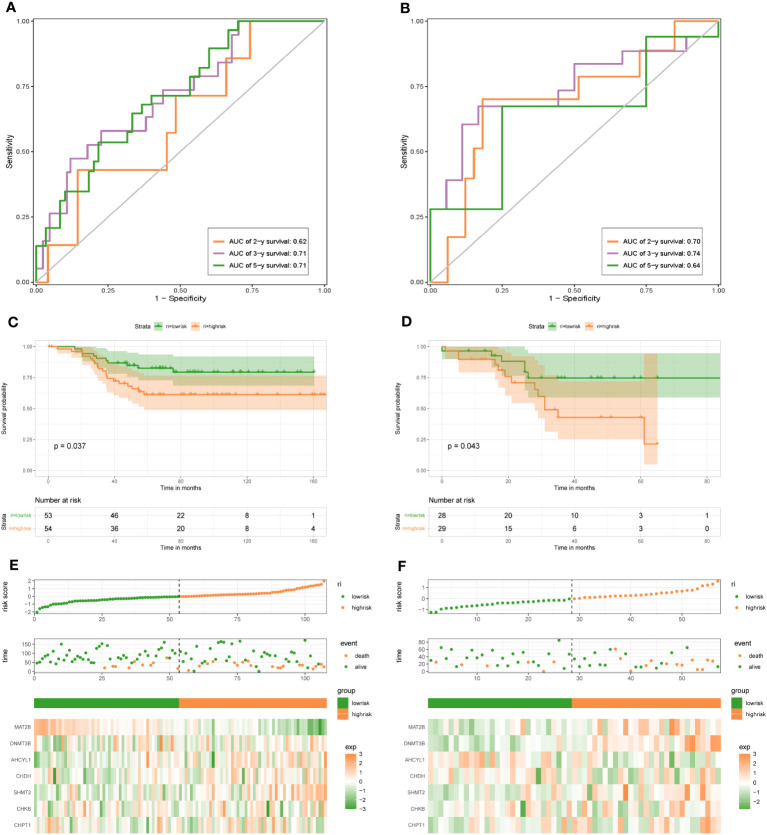
1C metabolism-related gene signature associated with the OS and DFS of BC patients. **(A)** The predictive value for the 2-y, 3-y, and 5-y OS in GSE58812 dataset. **(B)** The predictive value for the 2-y, 3-y, and 5-y DFS in GSE61304 dataset. **(C)** The OS between the high- and low-risk groups in GSE58812 dataset. **(D)** The DFS between the high- and low-risk groups in GSE61304 dataset. **(E)** The risk plot of the 1C metabolism-related genes signature in GSE58812 dataset. **(F)** The risk plot of the 1C metabolism-related genes signature in GSE61304 dataset.

To demonstrate the credibility of the prediction of the seven 1C metabolism-related genes, we conducted survival analyses and plotted ROC curves on the training and test cohorts, respectively. The AUCs for 2-, 3- and 5-year survival in the GSE20685 training set were 0.79, 0.76, and 0.78, respectively (**Figure 2A**), while in GSE88770, GSE58812, and GSE61304 the AUCs for 2-, 3- and 5- year were 0.84, 0.71 and 0.76; 0.62, 0.71 and 0.71; 0.70, 0.74 and 0.64, respectively (**Figures 2B**, **3A, B**). In addition, Kaplan-Meier analysis revealed that survival time was markedly shorter in the high-risk group than the other one (GSE20685: p <0.0001; GSE88770: p = 0.0031; GSE58812: p = 0.037; GSE61304: p = 0.043) (**Figures 2C, D**, **3C, D**). Furthermore, risk curves and scatter plots showed that mortality increases with risk scores in all four datasets (**Figures 2E, F**, **3E, F**). The heat maps also showed remarkable expression differences in seven prognostic genes between both groups (**Figures 2E, F**, **3E, F**).

### 3.3 Independent prognostic analysis and nomogram development

**Figure 4:**
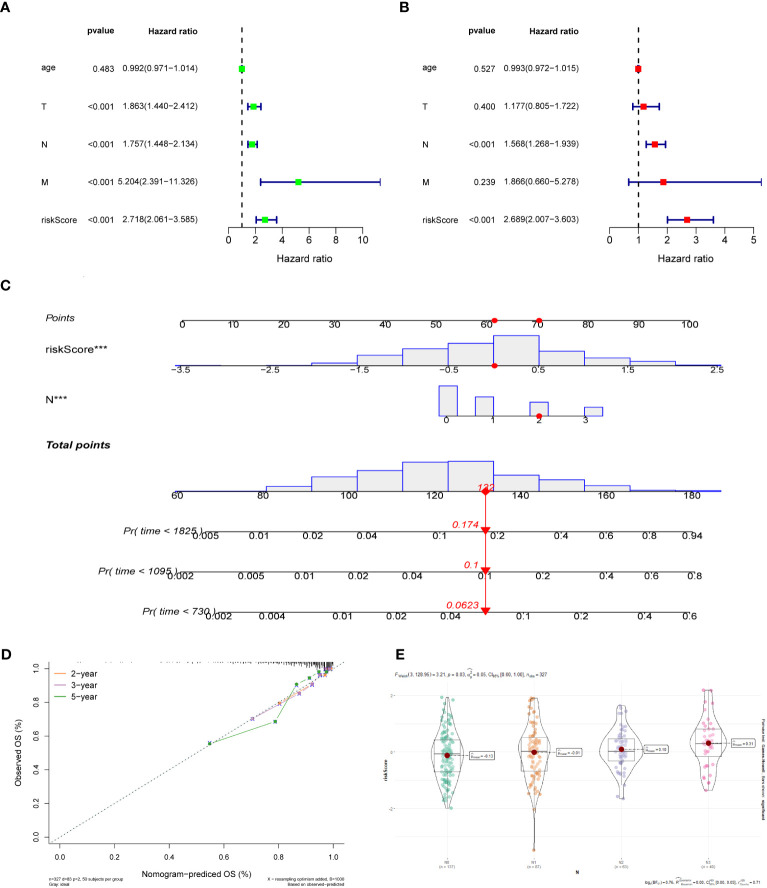
**(A)** Univariate Cox regression analysis of the risk score and clinical parameters. **(B)** Multivariate Cox regression analysis of the risk score and clinical parameters. **(C)** The construction of 2-, 3-, and 5-year OS predictive nomogram for patients of the GSE20685. **(D)** Calibration curves for the nomogram. **(E)** Distribution of risk scores in different N-stage in GSE20685 dataset.

For the training dataset GSE20685, in the univariate Cox analysis, TNM stage and risk score were closely linked to OS in BC patients (p < 0.001), whereas in multivariate Cox analysis, only N-stage and risk score were independent prognostic predictors (p < 0.001) (**Figures 4A, B**). Therefore, a nomogram was built to quantitatively predict individual OS at 2-, 3-, and 5-year based on independent prognostic markers (N-stage and risk score) (**Figure 4C**). Then, to verify its predictive effectiveness, calibration curves were plotted to confirm the consistency, which showed the desired predictive accuracy (**Figure 4D**). In summary, the nomogram can predict short- and long-term OS in BC patients, thus contributing to clinical management. Furthermore, the performance of the multivariate Cox model containing clinical characteristic information and risk score on the training and test datasets was shown in **Supplementary Figures 2A-H**. In addition, the N-stage was positively linked to risk score (**Figure 4E**), with a statistically significant difference (p < 0.05).

### 3.4 Mutation landscape analysis

**Figure 5:**
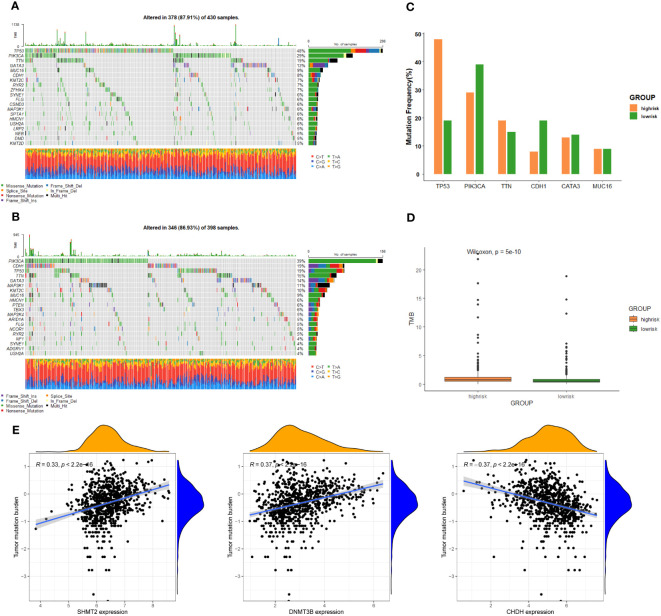
Genomic alterations between the high-risk group and low-risk group. **(A)** The landscape of mutation profiles in the high-risk group. **(B)** The landscape of mutation profiles in the low-risk group. **(C)** The six genes with the greatest variation in mutation frequency in the high-risk group and low-risk group. **(D)** The difference in tumor mutation burden between the high-risk group and low-risk group. **(E)** Correlation between the expression levels of target genes and tumor mutation burden.

To further investigate discrepancies in the genetic landscape between both groups, somatic mutation data from TCGA database of BC patients were used for analysis. In the high-risk group, TP53 had the highest mutation frequency at 48%, followed by PIK3CA, TTN, GATA3, MUC16, and CDH1 (**Figure 5A**). Correspondingly, in the low-risk group, the top six genes in terms of mutation frequency were PIK3CA, CDH1, TP53, TTN, GATA3, and MAP3K1 (**Figure 5B**). Meanwhile, the mutation frequency of the same gene differed considerably between groups (**Figure 5C**). TMB was higher in the high-risk group compared to the other group (**Figure 5D**). In addition, the expression of SHMT2 and DNMT3B was positively correlated with TMB, while the expression of CHDH was negatively correlated with TMB (P < 0.05) (**Figure 5E**), suggesting that these genes play a role in immunotherapy.

### 3.5 Gene set enrichment analysis

**Figure 6:**
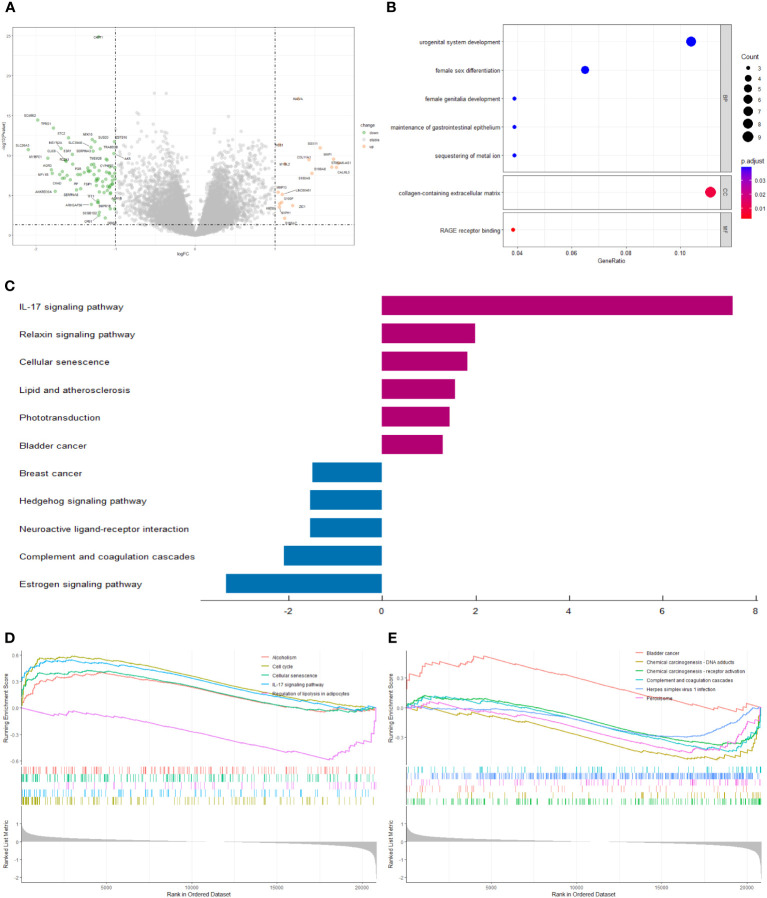
**(A)** The volcano plot of differential gene expression in high- and low-risk groups (|log FC| > 1 and p-value_ t < 0.05). **(B)** Bubble plot for GO enrichment analysis. **(C)** Two-way bar chart for KEGG enrichment analysis. **(D, E)** Results of GSEA in GSE20685 cohort.

We screened 98 differentially expressed genes (DEGs) by the “limma” variance approach, with 17 up-regulated as well as 81 down-regulated genes (**Figure 6A**). GO terms in biological process (BP), cellular component (CC), and molecular function (MF) that were significantly associated with these DEGs were represented in the bubble diagram (**Figure 6B**), suggesting that these DEGs were mainly related to urogenital system development, female sex differentiation, and collagen-containing extracellular matrix. Furthermore, we found that the IL-17 signaling pathway, relaxin signaling pathway, cellular senescence, lipid and atherosclerosis, phototransduction, and bladder cancer are up-regulated, while breast cancer, hedgehog signaling pathway, neuroactive ligand-receptor interaction, complement and coagulation cascades, and estrogen signaling pathway are down-regulated (**Figure 6C**). In addition, to identify the underlying biological signaling pathways for molecular discrepancies between both risk groups, we performed GSEA analyses (**Figures 6D, E**). The results indicated that in the high-risk group, pathways such as alcoholism, cell cycle, cellular senescence, IL-17 signaling pathway, and bladder cancer were significantly enriched, while pathways significantly enriched in the low-risk group included regulation of lipolysis in adipocytes, chemical carcinogenesis-DNA adducts, chemical carcinogenesis-receptor activation, the complement and coagulation cascades, herpes simplex virus 1 infection, and peroxisome.

### 3.6 Immuno-infiltration analysis

**Figure 7:**
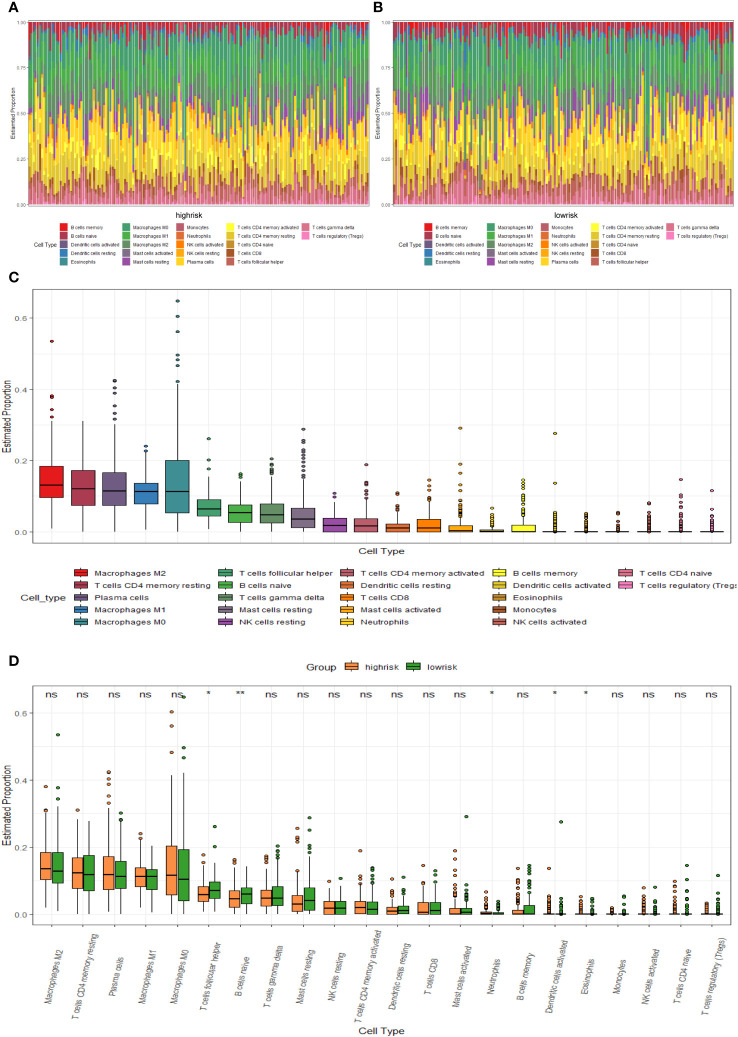
TME immune cell infiltration of BC samples from the GSE20685 cohort. **(A)** Heat map of the differences in immune cell distribution for each BC patient in high-risk group. **(B)** Heat map of the differences in immune cell distribution for each BC patient in low-risk group. **(C)** Histogram of the distribution of immune cells in all BC patients. **(D)** Differences in the distribution of immune cells in high- and low-risk groups.

We compared the levels of immune infiltration in the two risk groups, and the distribution of immune cells in both was reflected in **Figures 7A, B**. In addition, the overall immune infiltration in all BC samples in the training set was illustrated in **Figure 7C**. Further combined with the difference and correlation analysis, some immune cell subpopulations showed statistically significant differences between both groups. In particular, the infiltration levels of T follicular helper (Tfh) and B cells naive were lower in the high-risk group, whereas Neutrophils infiltration abundance was higher in the high-risk group (**Figure 7D**).

### 3.7 Immune checkpoints and immunotherapy research

**Figure 8:**
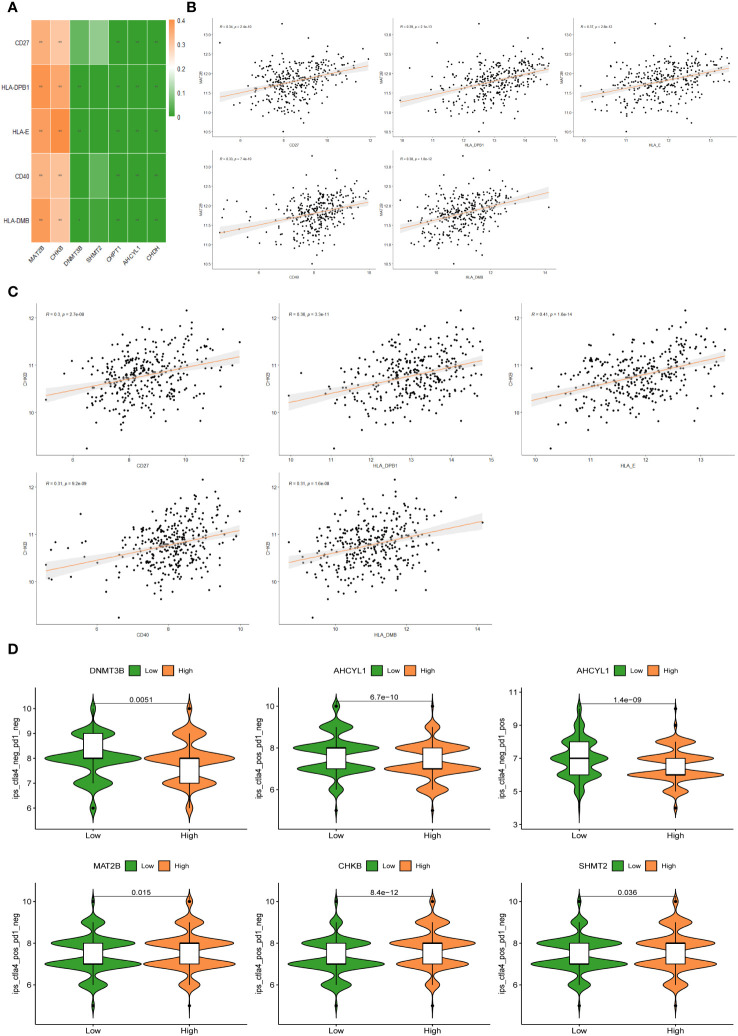
**(A)** Correlation heat map between 5 immune checkpoints and 7 1C metabolism-related genes. **(B)** Correlation between MAT2B expression levels and 5 immune checkpoints. **(C)** Correlation between CHKB expression levels and 5 immune checkpoints. **(D)** Differences in IPS reactivity between high and low expression levels of target genes.

The heat map of the association between 7 1C metabolism-related genes and 71 ICGs was displayed in **Supplementary Figure 3**. Among them, the 5 ICGs that were significantly correlated with the 1C metabolism-related genes were selected for redrawing the heat map (**Figure 8A**). In addition, MAT2B and CHKB are closely associated with immune checkpoints as shown in **Figures 8B, C**. Furthermore, BC patients with low expressions of DNMT3B and AHCYL1 had higher IPS, indicating that these patients had higher relative probabilities of responding to ICIs, whereas BC patients with high expressions of MAT2B, CHKB, and SHMT2 had higher relative probabilities of responding to ICIs (**Figure 8D**).

### 3.8 Drug sensitivity analysis

**Figure 9:**
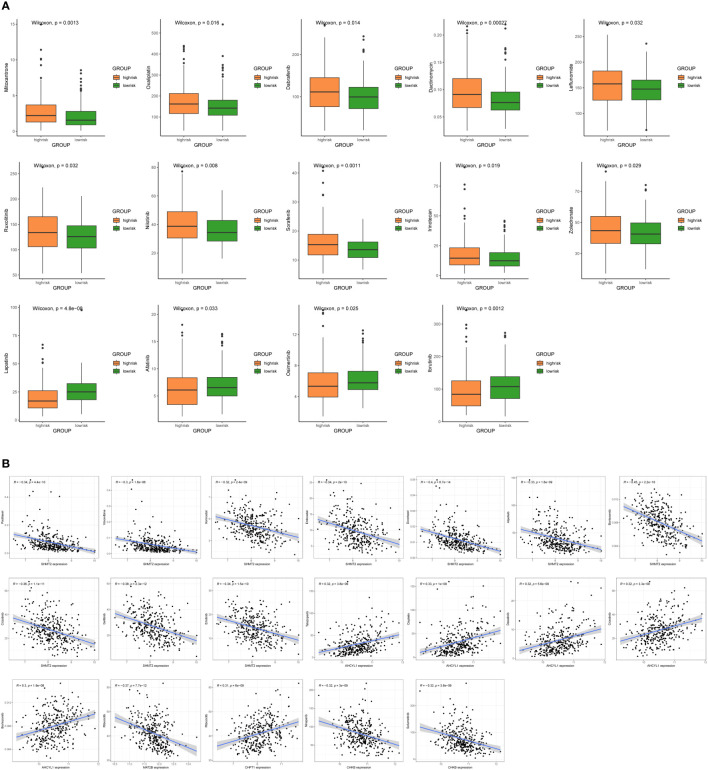
Sensitivity analysis of chemotherapeutic drugs and prediction of potential drugs. **(A)** Comparison of IC50 values for chemotherapy and targeted drugs in two groups. **(B)** Correlation analysis of 7 1C metabolism-related genes and potential drug sensitivity.

To improve the clinical utility of survival models in the management of BC, we calculated and compared IC50 values in two groups of patients, because IC50 values are inversely related to drug sensitivity. The results revealed that low-risk patients were more sensitive to Mitoxantrone, Oxaliplatin, Dabrafenib, Dactinomycin, Leflunomide, Ruxolitinib, Nilotinib, Sorafenib, Irinotecan, and Zoledronate. Meanwhile, high-risk patients were more sensitive to Lapatinib, Afatinib, Osimertinib, and Ibrutinib (P < 0.05) (**Figure 9A**). In addition, there are drugs targeting 7 1C metabolism-related genes available for the treatment of BC. We discovered a positive association between SHMT2 expression and sensitivity to Paclitaxel, Vinorelbine, Vorinostat, Entinostat, Docetaxel, Alpelisib, Bortezomib, Crizotinib, Gefitinib, and Erlotinib. The expression of AHCYL1 was negatively associated with Talazoparib, Cisplatin, Dasatinib, Crizotinib, and Bortezomib. The expression of MAT2B was positively related to Ribociclib. The sensitivity to Ribociclib was negatively linked to CHPT1 expression. CHKB expression was positively connected to Niraparib and Selumetinib (**Figure 9B**). With the above findings, the risk score can be used as a guide for medication administration in BC patients.

## 4 Discussion

BC has surpassed lung cancer as the most prevalent cancer in women (24). Despite improvements in its multidisciplinary treatment, BC remains the leading cause of death in female cancer patients (24, 25). Alterations in the metabolism of cancer cells are critical for tumor growth, and one of the most remarkable aspects of this metabolic reprogramming is the 1C metabolism (26). However, there is still a lack of studies on the 1C metabolism in BC patients. Therefore, our research aims to make an essential step in that direction.

We constructed a survival risk signature by 1C metabolism-related genes in this study, which performed well in both training and validation set cohorts. Furthermore, Kaplan-Meier analysis showed that two genes can be regarded as risk factors, including SHMT2 and DNMT3B, and five genes were identified as protective factors, including MAT2B, AHCYL1, CHDH, CHKB, and CHPT1. Moreover, the expression values of these genes were also measured in both groups. Two genes were up-regulated in high-risk patients, consisting of SHMT2 and DNMT3B, while the down-regulated genes included MAT2B, AHCYL1, CHDH, CHKB, and CHPT1, which was consistent with Kaplan-Meier analysis. Among them, SHMT2 is considered to be an important factor in the metabolism of serine and glycine of several cancer cell types (including BC) (27), which is crucial in the development of cancer cells, and high SHMT2 expression is linked to poor prognosis in BC (28). DNMT3B acts as a key player in breast tumorigenesis and development, and targeting DNMT3B may be a potential treatment for BC (29). Conversely, CHDH is an estrogen-regulated gene that is overexpressed in BC patients with good prognosis (30). As an enzyme related to methionine metabolism, MAT2B can act as a cancer suppressor gene in BC development (31). In addition, CHPT1, AHCYL1, and CHKB have been demonstrated to be associated with roles that lead to other cancers and affect patient outcomes, although relevant studies are scarce in BC (32–34).

Tumor-infiltrating immune cells are reported to be an essential component of TME and can be used to predict cancer prognosis (35). Hence, immune cells have been identified as a new cancer treatment target (36). Differences in TME between the two groups were examined using CIBERSORT. The results illustrated higher levels of infiltration of Tfh cells and naive B cells, and lower levels of infiltration of neutrophils in low-risk patients. Tfh cells are reported to be a subset of CD4^+^ helper T cells involved in the humoral response (37), whose role is to trigger B cells in the germinal center to differentiate into plasma cells secreting antibodies and memory B cells, which is the key to enhancing the immune response (38, 39). In addition, naive B cells are activated, proliferate, and differentiate into plasma cells and memory B cells, which are involved in antitumor immunity (40). Accordingly, we presume that low-risk patients might benefit more from immunotherapy. Moreover, neutrophils can produce immunosuppressive factors, such as transforming growth factor beta (TGF-β) and arginase1, effectively suppressing adaptive immunity (41), and release growth factors such as hepatocyte growth factor (42), which promote tumor progression. It has also been shown that a high neutrophil/lymphocyte ratio in the circulation is a poor prognostic factor in breast, liver, colon, and many other types of cancer (41, 43), which is consistent with our findings. However, the results did not demonstrate an effect of 1C metabolism on T cell activation. It has been reported that 1C metabolism contributes to purine and thymidine synthesis, allowing T cell proliferation and survival, whereas genetic inhibition of the metabolic enzyme SHMT2 impaired T cell survival and antigen-specific T cell abundance in culture and *in vivo*, respectively (44). The interaction of these factors may have led to the generation of such inconsistent results. It may also be due to differences in study design and clinical characteristics of the subjects.

In our study, the IL-17 signaling pathway was significantly enriched in KEGG and GSEA. The IL-17 family comprises six members (IL-17A to IL-17F) with distinct functions and sequence homologies (45). Their aberrant expression is closely linked to chronic inflammatory diseases and acts as an essential player in cancer immunity (46). A number of recent findings have elucidated the effect of the IL-17B/IL-17RB pathway in tumorigenesis. For example, in mice, IL-17B signaling via IL-17RB facilitates cancer cell survival, invasion, proliferation, and metastasis (47–50), whereas in humans, high expression of IL-17B and IL-17RB is linked to a poorer prognostic outcome in BC sufferers (48). In addition, the peroxisome and herpes simplex virus-1 infection were found to be enriched in the low-risk group. Peroxisomes are organelles that affect the growth and survival of cancer cells, and some cancer cells rely on lipolysis by peroxisomes for their energy needs (51). Oncolytic herpes simplex virus-1 infection increases anticancer activity by inducing apoptosis in adjacent cancer cells (52). The enrichment of these pathways is consistent with the finding that low-risk patients survived longer.

ICIs rebuild the anti-tumor immune response by blocking co-inhibitory signaling pathways, thereby promoting immune-mediated elimination of tumor cells (53). Although ICIs, particularly anti-CTLA4 and anti-PD-1 antibodies, have radically improved the prognosis of many cancers, especially advanced melanoma (54), they have been less effective in BC patients (55). This approach can be used to identify and screen patients who respond to treatment. Based on the research results, BC patients with high expression of MAT2B and CHKB may benefit from targeted therapy against immune checkpoints with increased expression, such as CD27, CD40, HLA-DPB1, HLA-E, and HLA-DMB. The results of immunotherapy analysis further proved the potential of these seven candidate genes as novel prognostic indicators and intervention targets for signature development. Therefore, for BC patients, using our 1C metabolism-related genes model to predict their response to ICIs can guide clinical treatment more concretely and effectively.

Although some positive results have been obtained, several limitations of our study remain. Since this is a retrospective study, data omissions and selection bias are inevitable. Secondly, our study is based on data from existing publicly accessible databases, so the results need to be further validated in large cohorts. Further, people in GSE61304 dataset have been followed for only 80 months while in other datasets individuals have been followed up for more than 150 months. This discrepancy in follow-up time can create a problem in the validation. Finally, in-depth characterization of the mechanism through *in vivo* or *in vitro* experiments is required.

## 5 Conclusion

In summary, seven 1C metabolism-related genes were identified, resulting in a risk score model that can accurately assess OS in BC patients. Individuals with low-risk scores have longer survival and are better able to benefit from immunotherapy. We believe that these seven genes should be used prospectively in BC patients to predict their prognosis and guide clinical chemotherapy and immunotherapy.

## Data availability statement

The datasets presented in this study can be found in online repositories. The names of the repository/repositories and accession number(s) can be found in the article/**Supplementary Material**.

## Author contributions

TZ: Writing – original draft. JL: Writing – review & editing. MW: Writing – review & editing. XL: Writing – review & editing. JQ: Writing – review & editing. HZ: Writing – review & editing.
