# Supplementary material for: Prognosis stratification and response to treatment in breast cancer based on one-carbon metabolism-related signature
Source: Front Oncol. 2024 Jan 4;13:1288909. doi: 10.3389/fonc.2023.1288909 (PMC10794736; doi:10.3389/fonc.2023.1288909)

Supplementary Material

# Supplementary Figures and Tables

## Supplementary Tables

**Supplementary Table 1.** 72 1C metabolism-related genes were retrieved from the MSigDB with the keyword "one-carbon metabolism".

|  | **NCBI (Entrez) Gene Id** | **name** | **Gene Description** |
| --- | --- | --- | --- |
| 1 | 102724560 | ENSG00000274276 | cystathionine-beta-synthase like |
| 2 | 1036 | CDO1 | cysteine dioxygenase type 1 [... |
| 3 | 10390 | CEPT1 | choline/ethanolamine phosphot... |
| 4 | 10400 | PEMT | phosphatidylethanolamine N-me... |
| 5 | 10768 | AHCYL1 | adenosylhomocysteinase like 1... |
| 6 | 1119 | CHKA | choline kinase alpha [Source:... |
| 7 | 1120 | CHKB | choline kinase beta [Source:H... |
| 8 | 1491 | CTH | cystathionine gamma-lyase [So... |
| 9 | 1757 | SARDH | sarcosine dehydrogenase [Sour... |
| 10 | 1759 | DNM1 | dynamin 1 [Source:HGNC Symbol... |
| 11 | 1788 | DNMT3A | DNA methyltransferase 3 alpha... |
| 12 | 200895 | DHFR2 | dihydrofolate reductase 2 [So... |
| 13 | 23743 | BHMT2 | betaine--homocysteine S-methy... |
| 14 | 2571 | GAD1 | glutamate decarboxylase 1 [So... |
| 15 | 2572 | GAD2 | glutamate decarboxylase 2 [So... |
| 16 | 257202 | GPX6 | glutathione peroxidase 6 [Sou... |
| 17 | 27232 | GNMT | glycine N-methyltransferase [... |
| 18 | 2729 | GCLC | glutamate-cysteine ligase cat... |
| 19 | 2730 | GCLM | glutamate-cysteine ligase mod... |
| 20 | 2876 | GPX1 | glutathione peroxidase 1 [Sou... |
| 21 | 2877 | GPX2 | glutathione peroxidase 2 [Sou... |
| 22 | 2878 | GPX3 | glutathione peroxidase 3 [Sou... |
| 23 | 2879 | GPX4 | glutathione peroxidase 4 [Sou... |
| 24 | 2880 | GPX5 | glutathione peroxidase 5 [Sou... |
| 25 | 2882 | GPX7 | glutathione peroxidase 7 [Sou... |
| 26 | 2936 | GSR | glutathione-disulfide reducta... |
| 27 | 2937 | GSS | glutathione synthetase [Sourc... |
| 28 | 29958 | DMGDH | dimethylglycine dehydrogenase... |
| 29 | 4143 | MAT1A | methionine adenosyltransferas... |
| 30 | 4144 | MAT2A | methionine adenosyltransferas... |
| 31 | 4524 | MTHFR | methylenetetrahydrofolate red... |
| 32 | 4548 | MTR | 5-methyltetrahydrofolate-homo... |
| 33 | 5130 | PCYT1A | phosphate cytidylyltransferas... |
| 34 | 51380 | CSAD | cysteine sulfinic acid decarb... |
| 35 | 5337 | PLD1 | phospholipase D1 [Source:HGNC... |
| 36 | 55224 | ETNK2 | ethanolamine kinase 2 [Source... |
| 37 | 55349 | CHDH | choline dehydrogenase [Source... |
| 38 | 55500 | ETNK1 | ethanolamine kinase 1 [Source... |
| 39 | 56994 | CHPT1 | choline phosphotransferase 1 ... |
| 40 | 570 | BAAT | bile acid-CoA:amino acid N-ac... |
| 41 | 5833 | PCYT2 | phosphate cytidylyltransferas... |
| 42 | 586 | BCAT1 | branched chain amino acid tra... |
| 43 | 587 | BCAT2 | branched chain amino acid tra... |
| 44 | 635 | BHMT | betaine--homocysteine S-methy... |
| 45 | 6470 | SHMT1 | serine hydroxymethyltransfera... |
| 46 | 6472 | SHMT2 | serine hydroxymethyltransfera... |
| 47 | 64902 | AGXT2 | alanine--glyoxylate aminotran... |
| 48 | 6647 | SOD1 | superoxide dismutase 1 [Sourc... |
| 49 | 6648 | SOD2 | superoxide dismutase 2 [Sourc... |
| 50 | 6649 | SOD3 | superoxide dismutase 3 [Sourc... |
| 51 | 7298 | TYMS | thymidylate synthetase [Sourc... |
| 52 | 9468 | PCYT1B | phosphate cytidylyltransferas... |
| 53 | 10588 | MTHFS | methenyltetrahydrofolate synthetase [Source... |
| 54 | 10797 | MTHFD2 | methylenetetrahydrofolate dehydrogenase (NA... |
| 55 | 10840 | ALDH1L1 | aldehyde dehydrogenase 1 family member L1 [... |
| 56 | 10841 | FTCD | formimidoyltransferase cyclodeaminase [Sour... |
| 57 | 123263 | MTFMT | mitochondrial methionyl-tRNA formyltransfer... |
| 58 | 1719 | DHFR | dihydrofolate reductase [Source:HGNC Symbol... |
| 59 | 1786 | DNMT1 | DNA methyltransferase 1 [Source:HGNC Symbol... |
| 60 | 1789 | DNMT3B | DNA methyltransferase 3 beta [Source:HGNC S... |
| 61 | 191 | AHCY | adenosylhomocysteinase [Source:HGNC Symbol;... |
| 62 | 23382 | AHCYL2 | adenosylhomocysteinase like 2 [Source:HGNC ... |
| 63 | 2346 | FOLH1 | folate hydrolase 1 [Source:HGNC Symbol;Acc:... |
| 64 | 25902 | MTHFD1L | methylenetetrahydrofolate dehydrogenase (NA... |
| 65 | 2618 | GART | phosphoribosylglycinamide formyltransferase... |
| 66 | 27430 | MAT2B | methionine adenosyltransferase 2B [Source:H... |
| 67 | 2745 | GLRX | glutaredoxin [Source:HGNC Symbol;Acc:HGNC:4... |
| 68 | 275 | AMT | aminomethyltransferase [Source:HGNC Symbol;... |
| 69 | 4522 | MTHFD1 | methylenetetrahydrofolate dehydrogenase, cy... |
| 70 | 4552 | MTRR | 5-methyltetrahydrofolate-homocysteine methy... |
| 71 | 471 | ATIC | 5-aminoimidazole-4-carboxamide ribonucleoti... |
| 72 | 6948 | TCN2 | transcobalamin 2 [Source:HGNC Symbol;Acc:HG... |

**Supplementary Table 2.** 79 Immune checkpoint genes retrieved from literature.

|  | **Gene symbol** | **Descriptions** |
| --- | --- | --- |
| 1 | ADORA2A | Adenosine A2a Receptor |
| 2 | BTLA | B And T Lymphocyte Associated |
| 3 | BTN2A1 | Butyrophilin Subfamily 2 Member A1 |
| 4 | BTN2A2 | Butyrophilin Subfamily 2 Member A2 |
| 5 | BTN3A1 | Butyrophilin Subfamily 3 Member A1 |
| 6 | BTNL3 | Butyrophilin Like 3 |
| 7 | BTNL9 | Butyrophilin Like 9 |
| 8 | C10orf54 | V-Set Immunoregulatory Receptor |
| 9 | CD160 | CD160 Molecule |
| 10 | CD209 | CD209 Molecule |
| 11 | CD226 | CD226 Molecule |
| 12 | CD27 | CD27 Molecule |
| 13 | CD274 | CD274 Molecule |
| 14 | CD276 | CD276 Molecule |
| 15 | CD28 | CD28 Molecule |
| 16 | CD40 | CD40 Molecule |
| 17 | CD40LG | CD40 Ligand |
| 18 | CD47 | CD47 Molecule |
| 19 | CD70 | CD70 Molecule |
| 20 | CD80 | CD80 Molecule |
| 21 | CD86 | CD86 Molecule |
| 22 | CD96 | CD96 Molecule |
| 23 | CEACAM1 | CEA Cell Adhesion Molecule 1 |
| 24 | CTLA4 | Cytotoxic T-Lymphocyte Associated Protein 4 |
| 25 | HAVCR2 | Hepatitis A Virus Cellular Receptor 2 |
| 26 | HLA-A | Major Histocompatibility Complex, Class I, A |
| 27 | HLA-B | Major Histocompatibility Complex, Class I, B |
| 28 | HLA-C | Major Histocompatibility Complex, Class I, C |
| 29 | HLA-DMA | Major Histocompatibility Complex, Class II, DM Alpha |
| 30 | HLA-DMB | Major Histocompatibility Complex, Class II, DM Beta |
| 31 | HLA-DOA | Major Histocompatibility Complex, Class II, DO Alpha |
| 32 | HLA-DOB | Major Histocompatibility Complex, Class II, DO Beta |
| 33 | HLA-DPA1 | Major Histocompatibility Complex, Class II, DP Alpha 1 |
| 34 | HLA-DPB1 | Major Histocompatibility Complex, Class II, DP Beta 1 |
| 35 | HLA-DQA1 | Major Histocompatibility Complex, Class II, DQ Alpha 1 |
| 36 | HLA-DQB1 | Major Histocompatibility Complex, Class II, DQ Beta 1 |
| 37 | HLA-DRA | Major Histocompatibility Complex, Class II, DR Alpha |
| 38 | HLA-DRB1 | Major Histocompatibility Complex, Class II, DR Beta 1 |
| 39 | HLA-DRB3 | Major Histocompatibility Complex, Class II, DR Beta 3 |
| 40 | HLA-DRB4 | Major Histocompatibility Complex, Class II, DR Beta 4 |
| 41 | HLA-DRB5 | Major Histocompatibility Complex, Class II, DR Beta 5 |
| 42 | HLA-E | Major Histocompatibility Complex, Class I, E |
| 43 | HLA-F | Major Histocompatibility Complex, Class I, F |
| 44 | HLA-G | Major Histocompatibility Complex, Class I, G |
| 45 | ICOS | Inducible T Cell Costimulator |
| 46 | ICOSLG | Inducible T Cell Costimulator Ligand |
| 47 | IDO1 | Indoleamine 2,3-Dioxygenase 1 |
| 48 | KIR2DL1 | Killer Cell Immunoglobulin Like Receptor, Two Ig Domains And Long Cytoplasmic Tail 1 |
| 49 | KIR2DL2 | Killer Cell Immunoglobulin Like Receptor, Two Ig Domains And Long Cytoplasmic Tail 2 |
| 50 | KIR2DL3 | Killer Cell Immunoglobulin Like Receptor, Two Ig Domains And Long Cytoplasmic Tail 3 |
| 51 | KIR2DL4 | Killer Cell Immunoglobulin Like Receptor, Two Ig Domains And Long Cytoplasmic Tail 4 |
| 52 | KIR2DL5A | Killer Cell Immunoglobulin Like Receptor, Two Ig Domains And Long Cytoplasmic Tail 5A |
| 53 | KIR2DL5B | Killer Cell Immunoglobulin Like Receptor, Two Ig Domains And Long Cytoplasmic Tail 5B |
| 54 | KIR2DS1 | Killer Cell Immunoglobulin Like Receptor, Two Ig Domains And Short Cytoplasmic Tail 1 |
| 55 | KIR2DS2 | Killer Cell Immunoglobulin Like Receptor, Two Ig Domains And Short Cytoplasmic Tail 2 |
| 56 | KIR2DS3 | Killer Cell Immunoglobulin Like Receptor, Two Ig Domains And Short Cytoplasmic Tail 3 |
| 57 | KIR2DS4 | Killer Cell Immunoglobulin Like Receptor, Two Ig Domains And Short Cytoplasmic Tail 4 |
| 58 | KIR2DS5 | Killer Cell Immunoglobulin Like Receptor, Two Ig Domains And Short Cytoplasmic Tail 5 |
| 59 | KIR3DL1 | Killer Cell Immunoglobulin Like Receptor, Three Ig Domains And Long Cytoplasmic Tail 1 |
| 60 | KIR3DL2 | Killer Cell Immunoglobulin Like Receptor, Three Ig Domains And Long Cytoplasmic Tail 2 |
| 61 | KIR3DL3 | Killer Cell Immunoglobulin Like Receptor, Three Ig Domains And Long Cytoplasmic Tail 3 |
| 62 | KIR3DS1 | Killer Cell Immunoglobulin Like Receptor, Three Ig Domains And Short Cytoplasmic Tail 1 |
| 63 | LAG3 | Lymphocyte Activating 3 |
| 64 | LGALS9 | Galectin 9 |
| 65 | PDCD1 | Programmed Cell Death 1 |
| 66 | PDCD1LG2 | Programmed Cell Death 1 Ligand 2 |
| 67 | PVR | PVR Cell Adhesion Molecule |
| 68 | SIRPA | Signal Regulatory Protein Alpha |
| 69 | TDO2 | Tryptophan 2,3-Dioxygenase |
| 70 | TIGIT | T Cell Immunoreceptor With Ig And ITIM Domains |
| 71 | TNFRSF14 | TNF Receptor Superfamily Member 14 |
| 72 | TNFRSF18 | TNF Receptor Superfamily Member 18 |
| 73 | TNFRSF4 | TNF Receptor Superfamily Member 4 |
| 74 | TNFRSF9 | TNF Receptor Superfamily Member 9 |
| 75 | TNFSF14 | TNF Superfamily Member 14 |
| 76 | TNFSF18 | TNF Superfamily Member 18 |
| 77 | TNFSF4 | TNF Superfamily Member 4 |
| 78 | TNFSF9 | TNF Superfamily Member 9 |
| 79 | VTCN1 | V-Set Domain Containing T Cell Activation Inhibitor 1 |

## Supplementary Figures

**Supplementary Figure 1.** The forest graph showed the results of stepwise multivariate Cox proportional hazards regression analysis.


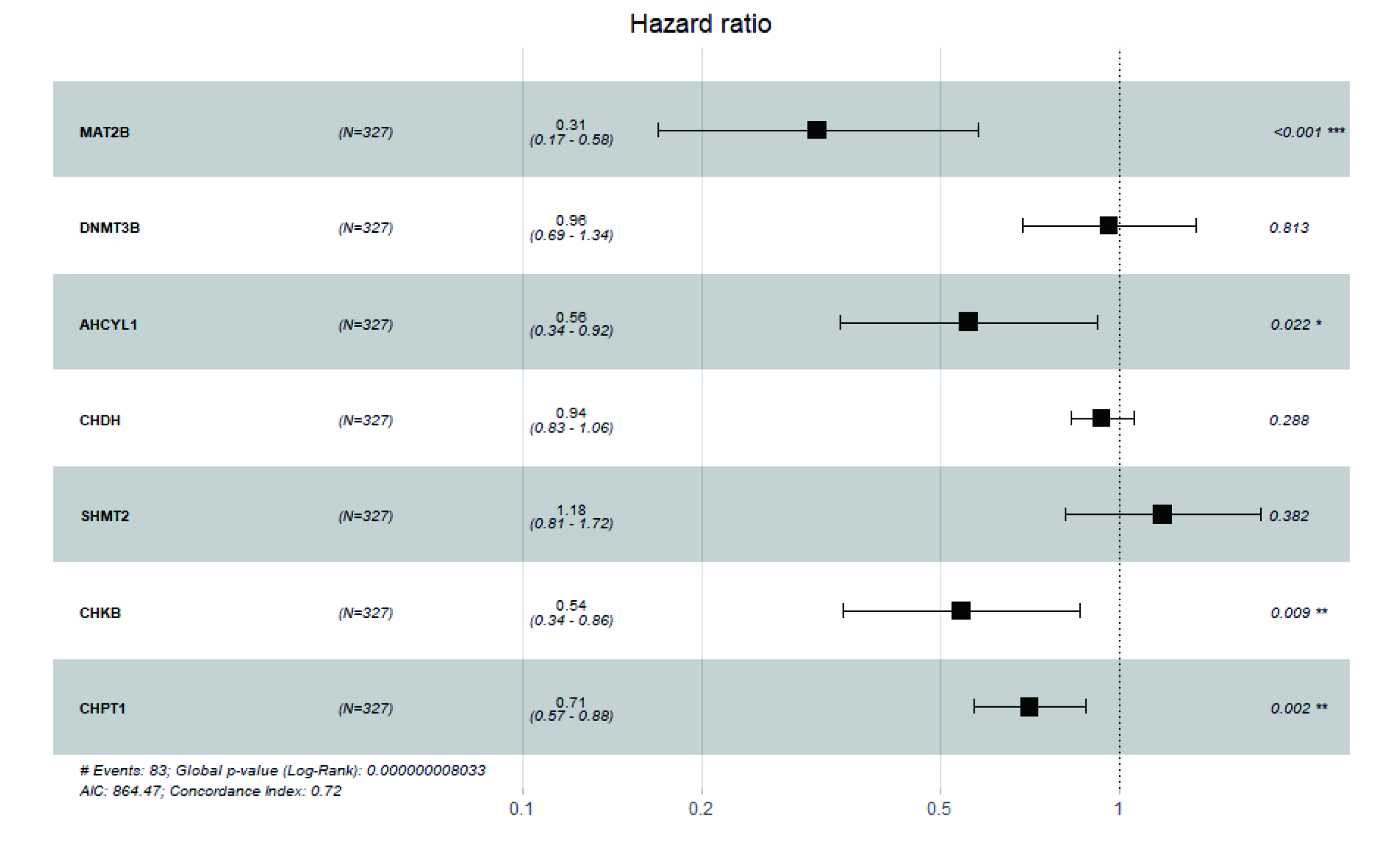


**Supplementary Figure 2.** Multivariate Cox regression analysis of the risk score and clinical parameters in **(A)** GSE20685, **(C)** GSE88770, **(E)** GSE58812, and **(G)** GSE61304 datasets. The predictive value for the multivariate Cox model in **(B)** GSE20685, **(D)** GSE88770, **(F)** GSE58812, **(H)** GSE61304 datasets.


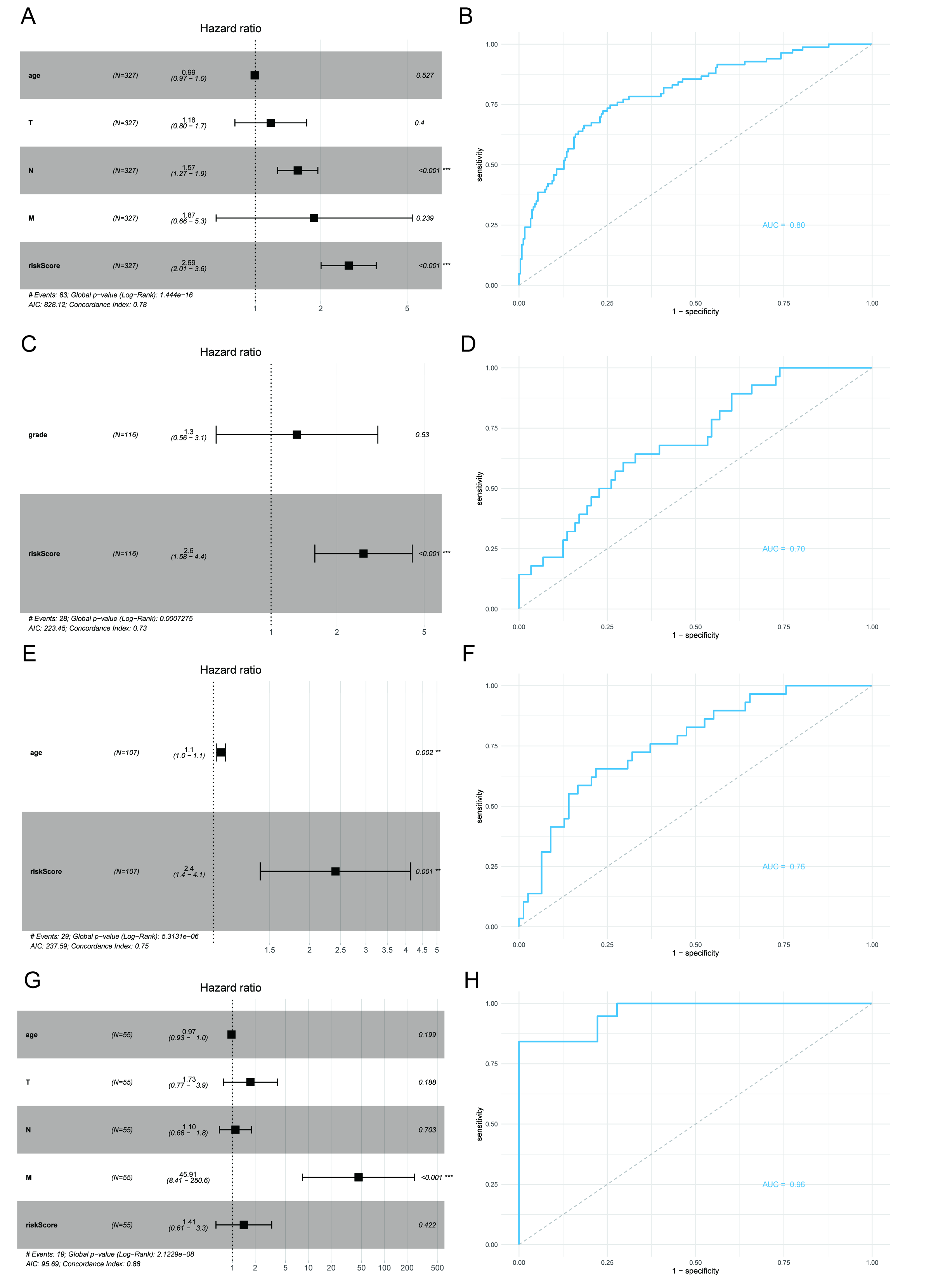


**Supplementary Figure 3.** The heat map of the correlation between 7 1C metabolism-related genes and 71 ICGs


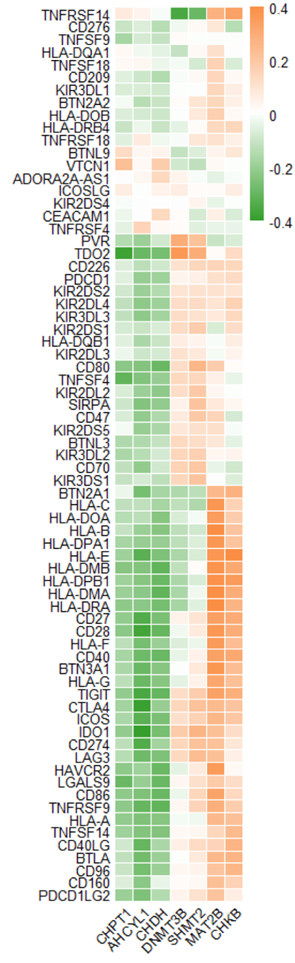

Supplement: Supplementary file 1 [file DataSheet_1.docx]
